# The Fabrication of Polymer-Based Curcumin-Loaded Formulation as a Drug Delivery System: An Updated Review from 2017 to the Present

**DOI:** 10.3390/pharmaceutics16020160

**Published:** 2024-01-24

**Authors:** Abul Kalam Azad, Joanne Lai, Wan Mohd Azizi Wan Sulaiman, Hassan Almoustafa, Salah Abdalrazak Alshehade, Vinoth Kumarasamy, Vetriselvan Subramaniyan

**Affiliations:** 1 Faculty of Pharmacy, University College of MAIWP International, Batu Caves, Kuala Lumpur 68100, Malaysia; drwanazizi@ucmi.edu.my; 2 Faculty of Pharmacy, MAHSA University, Jenjarom 42610, Selangor, Malaysia; joanne121700@gmail.com; 3 Faculty of Medicine, Universiti Malaya, Federal Territory of Kuala Lumpur, Kuala Lumpur 50603, Malaysia; hasan85@um.edu.my; 4 Faculty of Pharmacy & Bio-Medical Sciences, MAHSA University, Petaling Jaya 42610, Selangor, Malaysia; salah_alsh@outlook.com; 5 Department of Parasitology and Medical Entomology, Faculty of Medicine, Universiti Kebangsaan Malaysia, Jalan Yaacob Latif, Cheras, Kuala Lumpur 56000, Malaysia; 6 Pharmacology Unit, Jeffrey Cheah School of Medicine and Health Sciences, Monash University, Jalan Lagoon Selatan, Bandar Sunway 47500, Selangor, Malaysia; subramaniyan.vetriselvan@monash.edu; 7 Center for Transdisciplinary Research, Department of Pharmacology, Saveetha Dental College, Saveetha Institute of Medical and Technical Sciences, Saveetha University, Chennai 600077, Tamil Nadu, India

**Keywords:** curcumin, encapsulation, targeted drug delivery, colon cancer, absorption, bioavailability

## Abstract

Turmeric contains curcumin, a naturally occurring compound with noted anti-inflammatory and antioxidant properties that may help fight cancer. Curcumin is readily available, nontoxic, and inexpensive. At high doses, it has minimal side effects, suggesting it is safe for human use. However, curcumin has extremely poor bioavailability and biodistribution, which further hamper its clinical applications. It is commonly administered through oral and transdermal routes in different forms, where the particle size is one of the most common barriers that decreases its absorption through biological membranes on the targeted sites and limits its clinical effectiveness. There are many studies ongoing to overcome this problem. All of this motivated us to conduct this review that discusses the fabrication of polymer-based curcumin-loaded formulation as an advanced drug delivery system and addresses different approaches to overcoming the existing barriers and improving its bioavailability and biodistribution to enhance the therapeutic effects against cancer and other diseases.

## 1. Introduction

Turmeric, with the scientific name C. longa, comes from a rhizomatous medicinal plant in the Zingiberaceae family [1,2]. The plant contains curcuminoids, including curcumin, demethoxycurcumin, and bisdemethoxycurcumin, which provide therapeutic value [2,3]. Curcumin, also called diferuloylmethane, was first extracted from turmeric in 1815 and chemically identified in 1870 [3,4]. As the primary turmeric polyphenol, curcumin is widely used as a natural food additive for its aromatic, coloring, and antioxidant properties [4,5].

Curcumin exhibits many biological activities, including anti-inflammatory, anticancer, antiviral, antibacterial, antifungal, and immunomodulatory activities. It may also help treat arthritis, diabetes, obesity, and neurological disorders. Some studies suggest curcumin has cardioprotective, nephroprotective, and hepatoprotective properties [1,2,6]. Additionally, curcumin shows promise for preventing bone density loss like osteopenia, treating periodontitis and gingivitis, and reducing oral pain and swelling [2]. One study found curcumin could alleviate exercise-induced muscle soreness and inflammation, improving performance and recovery in active people. Although curcumin has numerous therapeutic effects, most are derived from its anti-inflammatory and antioxidant activities [5].

Curcumin is a lipophilic substance that has poor solubility in aqueous solutions [4,5]. Its absorption into GIT is inadequate, and it also has been proven that curcumin has a rapid metabolism and removal rate; subsequently, the serum level of free curcumin is low, and its bioavailability is extremely poor [4,5,7]. The tissue distribution of curcumin to its target action site is also found to be inefficient [8]. Hence, this limits the clinical application of curcumin in various diseases. Curcumin is unstable under alkaline conditions, where it will degrade rapidly and eventually cause low bioavailability [4]. Therefore, various approaches are being investigated to optimize its bioavailability and stability; for instance, microencapsulation is used to decrease its diameter and increase its surface area to drug ratio; thus, it improves dissolution rate and increases free curcumin levels in plasma [3]. This review summarizes past and current efforts to overcome problems related to curcumin delivery and suggests possible future directions with the most recent research findings.

### 1.1. Antioxidation and Anti-Inflammatory Actions of Curcumin

The chemical structure of curcumin, including its phenyl rings with hydroxyl and o-methoxy groups, 1,3-diketone system, and carbon–carbon double bonds, enables its potent antioxidant activity [4,9]. In particular, the phenolic structure has demonstrated antioxidant effects in multiple in vivo and in vitro studies. Curcumin regulates several antioxidant enzymes, such as reductase, catalase, glutathione peroxidase, and superoxide dismutase. By enhancing these enzymes’ activity, curcumin prevents lipid peroxidation [3,4,9]. It also upregulates glutathione S-transferase and its messenger RNA [4]. Additionally, curcumin suppresses reactive oxygen species-generating enzymes like xanthine oxidase, cyclooxygenase, and lipoxygenase enzymes, inhibiting free radical production. It also acts as a free radical scavenger for hydrogen peroxide, superoxide, and nitric oxide radicals [3,4,5]. Due to its lipophilic nature, curcumin can scavenge peroxyl radicals like vitamin E, a common antioxidant [5,9]. However, studies show that curcumin’s antioxidant activity is ten times more potent than vitamin E [2,4,10]. These mechanisms underlie curcumin’s use in pharmaceuticals and medicine.

Studies show oxidative stress and inflammation share common pathological processes, with one potentially instigating the other. For example, accumulated reactive oxygen species (ROS) may activate intracellular signaling cascades that promote proinflammatory gene expression, triggering inflammation [5,11]. Additionally, curcumin can regulate nicotinamide adenine dinucleotide phosphate oxidase levels, exerting antioxidant effects that reduce inflammation [9,11]. Curcumin’s antioxidant activity may thus help manage several inflammatory diseases [9]. Researchers attribute curcumin’s anti-inflammatory action primarily to its ability to inhibit inflammatory cascades and mediators like tumor necrosis factor alpha (TNF-α) and nuclear factor-kappa B (NF-κB) [5,12]. TNF-α is a major inflammatory mediator in various diseases, while NF-κB can modulate cell inflammation and apoptosis by activating proinflammatory cytokines and the inflammation cascade [3,9,12]. By acting on peroxisome proliferator-activated receptor gamma and inhibiting the NF-κB pathway, curcumin directly suppresses the NLRP3 inflammasome, an intracellular complex involved in inflammatory disease development [11]. It also downregulates activator protein-1, monocyte chemoattractant protein-1, macrophage inflammatory protein-1 alpha, C-reactive protein, and prostaglandin E2, further suppressing inflammation [10,11,12,13].

As the authors of [11] have found, curcumin’s ability to regulate immune cells benefits inflammatory disease treatment. It primarily targets dendritic cells, T-helper 17 (Th17) cells, and regulatory T (Treg) cells. Th17 cells drive inflammation by producing interleukin-17, -22, and -23. In contrast, Treg cells suppress inflammation. An imbalance between Th17 and Treg cell numbers and function can cause abnormal immune responses and inflammation. By blocking the IL-23/Th17 pathway, curcumin limits Th17 cell differentiation and controls Th17/Treg balance, mitigating inflammation [11]. It also suppresses enzymes intrinsically tied to inflammation and carcinogenesis like 5-LOX and COX-2 [9,12].

### 1.2. Anticancer Activity of Curcumin

Free radical generation and oxidative damage play a significant role in cancer development, particularly in damaging DNA and lipid peroxidation. Through its free radical scavenging, curcumin may aid cancer prevention [4,6,7]. It also suppresses NF-κB and signal transducer activation, along with the signal transducer and activator of transcription (STAT)-3 pathway activation, delaying cancer onset [6,10]. Additionally, curcumin inhibits angiogenic cytokines like IL-1, IL-6, and IL-23, hindering angiogenesis and carcinogenesis in some cancers [4,9]. Studies show it stops growth and the invasion of various cancer types by suppressing inflammatory mediators involved in early-stage cancer, including LOX-2, COX-2, AP-1, TNF-α, epidermal growth factor receptor (EGFR), and human epidermal growth factor receptor-2 (HER2), given the close anti-inflammatory and antitumor links. It also induces apoptosis in cancerous cells via the p53-dependent pathway. Activating the tumor suppressor p53 downregulates antiapoptotic genes while overexpressing proapoptotic genes, preventing proliferation and causing cell death at the G2 cell cycle phase pathway [6,7,9].

Additionally, curcumin upregulates miR-34a while suppressing miR-21 and miR-130a, triggering cell cycle arrest at the G2-mitotic phase, contributing to tumor suppression in colorectal cancer (CRC) [7,9,14,15,16]. It also suppresses miR-17-5p, miR-20a, and miR-27a; activates zinc finger and BTB domain-containing (ZBTB)-4 and ZBTB10; and inhibits specificity protein (Sp) transcription factors. This inhibits cancer cell growth and induces apoptosis. EGFR downregulation is key for CRC therapy, suppressing proliferation, invasion, and metastasis [7]. By reducing EGFR expression through lower early growth response (EGR) factor-1 activity, curcumin halted proliferation in Caco-2 and HT29 colon cancer cell lines. Its ability to disrupt the cell cycle and accelerate cell death may inhibit CRC progression. It also inhibited cyclin D1 and disrupted the G1 cell cycle phase in HCT-116 cancer cells [3,6] At high doses (>3 μM), curcumin functions as an alkylating agent in CRC, preventing cancer cell multiplication while sparing healthy cells.

A small subpopulation of cancer stem cells (CSCs) contributes to drug resistance in colon cancer cells [7]. Overcoming tumor recurrence is therefore a critical challenge in colorectal cancer (CRC) management. Studies show that curcumin can reverse drug resistance pathways and increase chemotherapy-resistant cell treatment susceptibility by regulating CSC signaling pathways, reducing the involved protein expression, and enhancing medication efficacy at various levels [6]. Evidence indicates that curcumin and its analogs act as potent chemotherapeutics and chemosensitizers on CSCs by modulating miRNAs [7]. Overall, these findings imply that curcumin may be useful for both CRC prevention and treatment.

### 1.3. Safety and Pharmacokinetic Issues Faced by Curcumin

Curcumin’s significant biological activity makes it an ideal precursor molecule for upcoming chemotherapy drugs against diseases, including advanced colorectal cancer (CRC). High oral doses are well tolerated, with minimal adverse effects, primarily some itching and gastrointestinal problems like flatulence, diarrhea, and constipation [4]. A phase 1 trial in which 25 participants received up to 8 g of curcumin daily for three months showed no toxicity signs. However, curcumin’s pharmaceutical clinical use is hampered by its low serum-free concentration, extremely poor bioavailability, and limited tissue distribution to target sites. This stems from low aqueous solubility, inadequate absorption, rapid metabolism, and high elimination [1,4,5]. As a strong lipophilic flavonoid, curcumin is stable in acidic conditions like the stomach. However, it degrades quickly in alkaline environments and has poor photostability, producing degradation products like vanillin and ferulic acid [4,10,11]. Due to instability at alkaline pH, it has a short 10 min half-life at physiological pH 7.4 [1].

Oral curcumin undergoes extensive first-pass metabolism in the liver, causing rapid conversion into inactive water-soluble metabolites. This reduces its absorption and bioavailability [3,4,11]. Once in circulation, it is reduced to hydrocurcumin and then conjugated with glucuronide and sulfate, forming inactive conjugates. This leaves a negligible amount of curcumin detected in tissues. Consequently, curcumin concentrations in both target tissues and blood serum are extremely low or undetectable—much less than needed to inhibit most anti-inflammatory targets [2,4,8,9,11]. Clinical studies using 8 g of oral curcumin daily showed rapid metabolism, resulting in low levels of free curcumin plasma, at 2.5 μg/L [7]. Moreover, 75% of 1 g of oral curcumin was excreted unchanged in rats, indicating poor absorption. No detectable blood or plasma curcumin was found in colorectal cancer patients taking 0.02 g of curcuminoids, 0.2 g of curcumin essential oil, and oral curcumin for 29 days, although significant fecal curcumin and curcumin sulfate were detected [7].

## 2. Approaches to Overcoming Existing Barriers of Curcumin

Optimizing oral curcumin’s bioavailability and stability directly impacts its plasma concentration and therapeutic benefits. The overarching principle for overcoming issues like rapid metabolism is improving bioavailability. This can be achieved by formulating a pH-sensitive colon-targeted drug delivery system (CTDDS) to preserve and release an adequate amount of curcumin at the preferred colon target site [17]. CTDDS provides prolonged retention time, decreased upper stomach absorption, and reduced first-pass metabolism. Consequently, properties like bioavailability and stability are enhanced, while dosing frequency is reduced [1,12]. Due to the small size of CTDDS, curcumin particles readily diffuse in the colon without irritation. Simultaneously, keeping serum concentrations low reduces adverse reactions, further improving patient compliance [17]. Microencapsulation, producing 0.5–1 mm diameter microbeads, is an attractive CTDDS method [17,18,19]. It also improves oral curcumin’s sensory experience by eliminating the bitter taste without altering its nature [20]. Studies show that micronized curcumin has a nine-fold higher bioavailability than unformulated curcumin due to the increased surface-area-to-drug ratio with a smaller diameter, improving the dissolution rate and bioavailability. Notably, 0.5 g of micronized curcumin led to a significant plasma level in humans, at 0.6 μg/mL, a remarkable outcome for curcumin [3]. The hydrogen donor site, α,β-unsaturated β-diketone moiety, is believed to be the breakdown point during curcumin hydrolysis and degradation in water [2]. Binding curcumin’s diketo reaction site with proteins, lipids, polymers, or macromolecules improves its solubility in water by protecting the site from hydrolysis. Other approaches to improving chemical stability include the synthetic modifications of oxidation sites like phenolic OH and enolic OH, and encapsulation in lipids [2].

Using nanosized particles to deliver curcumin to tumor sites either alone [21] or more likely in conjunction with conventional chemotherapeutic agents is a common approach in cancer-targeting strategies, with investigators searching for synergistic combinations and a reduction in side effects or studying how to overcome drug resistance with different chemotherapeutics like platinum compounds [22] in nonsmall cell lung cancer (NSCLC) and colon cancer and paclitaxel [23] in breast cancer. Injectable nanoparticles delivering curcumin to tumors have to be coated with a hydrophilic coating, almost exclusively composed of polyethylene glycol (PEG), and they provide a wide array of options for positive targeting techniques [24].

### 2.1. Microencapsulation and Nanoencapsulation in Polymeric Materials

Any drug delivery system should aim to achieve and maintain the target therapeutic drug concentration at the site in the body. This can be accomplished using a multiparticulate dosage form like microbeads or nanoparticles, which are divided into smaller subunits that each have the desired properties [19]. Microencapsulation is an attractive, advanced process commonly used to produce controlled drug release systems [19,25]. It involves the permanent or temporary coating of an unstable active substance with a polymeric or nonpolymeric material. This forms small spheres with diameters ranging from one to several hundred micrometers, enabling targeted and controlled drug release under specific circumstances [20,25,26,27].

The encapsulation of curcumin in polymer-based particulates is not without limitations. The main ones include the lack of mature methods for industrial scale-up, shelf stability issues for many formulations, difficulties in coencapsulating more hydrophilic drugs [28], the lack of sufficient toxicology data for most polymers being investigated, high immunogenicity for older generations of particles and high costs [29].

According to several studies, microencapsulation protects sensitive core materials from degradation under harsh conditions like heat, oxygen, acids, or alkalis by incorporating them within a protective wall [20,25,27]. It enables controlled or sustained drug release at a particular location in the body over time and under desired environmental conditions. This preserves an adequate drug amount to have the intended effect at a specific site [19,26,27]. Subsequently, core substance stability is improved, along with enhanced efficiency in factors like dose-dependent antioxidant and anti-inflammatory effects. Bioavailability is also boosted, reducing dosing frequency and dose amount while maintaining uniform delivery [19,20,25,30]. The coating material type is crucial in this regard [27]. Additionally, microencapsulation enhances the sensory experience of medication by eliminating unpleasant tastes, aromas, or odors like bitterness without altering the drug’s nature [20] Azad et al., 2020 [26]. As noted by [20], microencapsulation also aids core substance solubility and improves permeability.

### 2.2. Microencapsulation and Nanoencapsulation Techniques

There are various microencapsulation techniques, classified into three main groups: physical, chemical, and physicochemical methods. Physical methods include spray drying, spray chilling, fluidized bed coating, centrifugal extrusion, solvent evaporation, and supercritical fluid precipitation [18,25]. Chemical methods involve in situ interfacial polymerization and molecular inclusion complexation. Physicochemical techniques include micro- and nanoparticle preparation techniques that depend on high sheer homogenization like emulsion solvent displacement technique, as well as low energy techniques dependent on the Ouzo effect (nanoprecipitation) and micelle formation methods [28]. In addition to conventional techniques that have been extensively discussed elsewhere [31,32], novel approaches are under trial like coacervation, ionotropic gelation, and sol-gel encapsulation [18,25]. The choice of method depends on the core substance and encapsulant’s chemical and physical properties, along with the desired product characteristics and morphology [20,27]. Using different methods can result in variations in capsule size, shape, hygroscopicity, lipophilicity, surface tension, and thermal behavior [27]. The primary determinants influencing drug release are the encapsulated drug type, encapsulant characteristics, and drug-to-encapsulant ratio, as well as their interaction [27]. Some key factors to consider in selecting the optimum microencapsulation technique include production scale, cost-effectiveness, reproducibility, and mild processing conditions to preserve the activity of sensitive compounds like curcumin. Evaluating the advantages and limitations of each method based on these factors can help identify the ideal approach for a particular application. The goal is to select a technique that allows for the development of an encapsulated product with the desired properties to effectively deliver curcumin at the target site. Table 1 outlines the main encapsulation techniques for curcumin nanoparticles. Each method has relative advantages and limitations that must be balanced based on the specific formulation goals, as previously discussed. Overall, optimized encapsulation strategies are crucial to overcoming the poor bioavailability and chemical instability of curcumin and fully harnessing its therapeutic potential.

#### 2.2.1. Spray Drying

Spray drying is a microencapsulation method where the core material is suspended or dissolved in a polymer solution to form a feed solution. The feed solution is then atomized to produce a mist within a chamber. Hot air is added which dries the mist into a powder [20,27]. The resulting powder has a range of particle sizes depending on variables like feed solution properties and operating conditions [27]. As noted by [20,25,27], the key benefits of spray drying are its affordability, flexibility, and suitability for diverse materials. This method has a high encapsulation loading capacity and is easily scalable. The short dryer contact time also enables the handling of labile materials [20]. However, the high temperatures used may degrade active ingredients. For example, hot air was found to increase omega-3 fatty acid powder oxidation susceptibility, shortening shelf life [25,27].

For heat-sensitive compounds like curcumin, the high temperatures involved in spray drying could lead to activity loss. However, measures can be taken to mitigate this, such as using cold water in the atomization process or adding antioxidants. The affordability, scalability, and suitability for fragile substances make spray drying an attractive microencapsulation approach for curcumin. The proper optimization of operating parameters could lead to the production of an encapsulated curcumin powder with enhanced stability and bioavailability compared to unencapsulated curcumin.

#### 2.2.2. Spray Chilling

Spray chilling, also known as spray cooling, is a microencapsulation method similar to spray drying in processing. The main difference is that cold air is used throughout the chilling process instead of hot air [27]. In spray chilling, the core substance and polymer are atomized together into a mist. Cold air introduction solidifies the microdroplets, producing microencapsulated powder. As stated by [27], spray chilling has tremendous potential for industrial production scale-up. However, research shows that spray-cooled microcapsules are unstable and may expel the core material during storage.

For heat-labile compounds like curcumin, spray chilling offers a significant advantage over spray drying by avoiding high temperatures that could degrade activity. The cold air preserves curcumin’s instability while enabling encapsulation. Spray chilling also shares the scalability benefits of spray drying. However, the storage instability issue reported for some spray-chilled microcapsules needs consideration. Proper polymer selection and the optimization of operating parameters could potentially improve stability. However, evidence of spray-chilled curcumin microcapsule stability during storage would need evaluation. If stable formulations can be developed, spray chilling presents a promising microencapsulation technique for curcumin delivery. The cold-processing temperatures ensure preserved bioactivity, while the industrial scalability facilitates translation and widespread use.

#### 2.2.3. Fluidized Bed Coating

**Figure 1:**
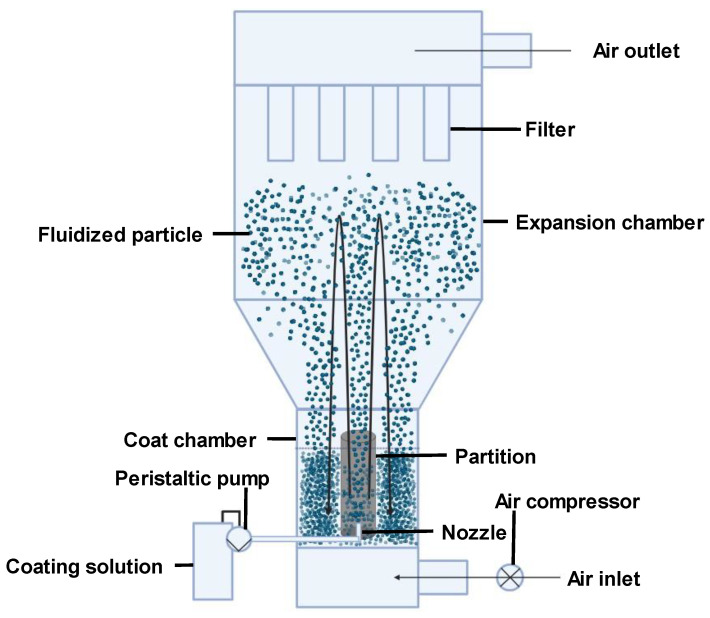
An illustration of the fluidized bed technique (bottom spray).

Fluidized bed coating is a microencapsulation technique where the coating material is sprayed over a fluidized core material [27]. The core is fluidized via air application. The coating can be applied through top spray, bottom spray (most common technique Figure 1), or tangential spray. Coating effectiveness depends on factors like the coating feed rate, atomization pressure, incoming air temperature, and velocity [27]. According to [33], fluidized bed coating offers advantages like easy manageability due to stable conditions and temperature runaway prevention through resistance to rapid temperature changes. It is useful for both large and small scales and enables continuous operation. However, this technique can only accommodate certain particle types and sizes. Its disparate flow patterns are also difficult to predict. Therefore, scaling up from small to industrial scales often poses challenges due to the complex behavior. Particle breakup is frequently observed as well. Particle collisions also cause pipe and vessel wall deterioration [33].

For heat-sensitive curcumin, fluidized bed coating enables uniform microcapsule coating under mild conditions that preserve bioactivity. Continuous operation and scalability are also beneficial for large-scale production. However, the particle size and type restrictions could limit encapsulation options, and particle collisions may damage curcumin. Proper polymer and operating parameter selection could mitigate these issues. For instance, using a polymer with cushioning properties could protect curcumin from damage during collisions. Fluidized bed coating presents a mild and scalable approach for curcumin microencapsulation, though particle restrictions and the potential for collisions require consideration.

#### 2.2.4. Coacervation Technique

Coacervation is a physicochemical microencapsulation method involving the formation of a uniform polymeric layer around the core substance. The core and polymer are mixed into an immiscible solution. Changes in pH, temperature, or ionic strength alter the polymer’s physicochemical properties, causing phase separation. Coacervates—small dense polymer droplets—are formed. Enclosing the core in these coacervates creates microcapsules. According to [27], coacervate formation stems from an electrostatic interaction between the two aqueous media. Coacervation is typically used for lipophobic molecule encapsulation. However, its usage is constrained as it functions optimally only within narrow pH ranges, and with certain electrolyte and colloidal solutions [27].

**Figure 2:**
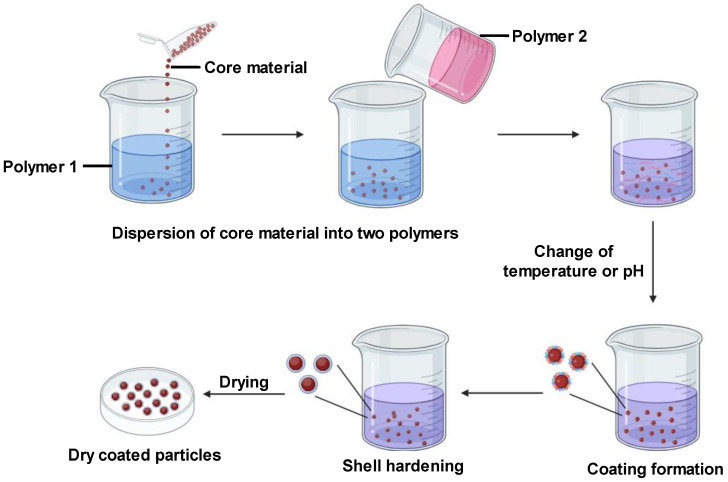
Complex coacervation technique for microencapsulation.

There are two types of coacervation: simple and complex. Simple coacervation uses one polymer like alginate. Sodium alginate is dissolved in water, and then the core material is added to the emulsion formed. This is released as droplets into a gel-forming medium like calcium chloride. The ionic reaction between sodium alginate and calcium chloride forms the insoluble polymer calcium alginate [27]. Complex coacervation involves multiple polymers, for instance, alginate and gelatin. Alginate and gelatin are solubilized in water at basic and acidic pH, respectively, generating negative and positive charges. The core material is added to the alginate solution and homogenized well. The gelatin and alginate phases are thoroughly mixed at increased temperature until a reaction is observed. This produces an insoluble polycationic–polyanionic polymer around the core [27]. For curcumin delivery, coacervation enables mild aqueous encapsulation that maintains stability. Simple coacervation with a suitable polymer could allow for tuned release. However, the constraint to narrow pH/electrolyte ranges may limit applications. Complex coacervation (Figure 2) provides more tailoring options with polymer combinations but needs extensive optimization. Overall, coacervation is a promising approach for stable aqueous curcumin encapsulation if optimal polymers and conditions are identified.

#### 2.2.5. Ionotropic Gelation Method

Due to its simplicity, ionotropic gelation has received the most attention for microbead creation compared to other techniques [19]. Its flexibility to generate a broad particle size range; medium-to-high drug encapsulation efficacy; and the use of biocompatible, biodegradable polymers also promote its usage [34]. This technique has frequently led to the production of naturally occurring, water-soluble polymeric nanoparticles with great control over bioactive ingredient release through polymer relaxation [34]. This method leverages an ionic polymer’s ability to crosslink with opposing ions to form a sustained-release hydrogel, as noted by [19]. Unlike simple monomeric ions, polyanion–cation interactions cannot be fully explained by electroneutrality. The three-dimensional structure and presence of the other group determine anions’ cation conjugation capacity, and vice versa. Research shows that microbeads prepared with this method have improved bioavailability and lead to controlled oral medication release, reducing dosing frequency [19]. Ionotropic gelation is divided into internal and external gelation based on the crosslinker ion source.

For curcumin, ionotropic gelation offers a simple, mild approach using biocompatible polymers to improve stability and bioavailability (Table 2). Tunable crosslinking enables sustained release customization. Internal gelation where the crosslinker is present in the initial mixture may better preserve curcumin during encapsulation. The method’s flexibility and control make it well suited for optimizing curcumin delivery through polymeric microbead encapsulation.

In internal gelation, the crosslinker ion is generated “in situ” within the polymer solution. Crosslinker cations come from insoluble metal salts like barium carbonate or calcium carbonate. Lowering the solution pH solubilizes the metal salt, releasing the metal ion internally [19]. In external gelation, crosslinker ions positioned externally come from a metal ion solution. The drug-containing polymer solution is extruded with a needle into the metal ion solution under gentle stirring. Gelation occurs instantly when the polymer drop contacts the metal ions, forming self-sustained beads. The beads are cured in the gelation medium before retrieval and drying. Rapid crosslinking ion diffusion into the partly gelled beads causes external gelation [19].

Ionotropic gelation has several advantages over conventional techniques, chiefly its simplicity and affordability due to the lack of complex machinery, aqueous solvents, and short processing times, as indicated in [34]. Additionally, the reversible electrostatic reaction-induced physical crosslinking prevents potential toxicity and other undesirable biomedical consequences, unlike chemical crosslinking [34]. However, poor mechanical stability is a drawback requiring further improvement [34].

For curcumin encapsulation, ionotropic gelation offers a simple, mild approach with short processing times that maintains stability. Internal gelation may better preserve curcumin by initially incorporating the crosslinker. The mechanical stability issues could be addressed through polymer modifications (Table 3) or gentle downstream processing. Overall, the method’s advantages make it promising for optimized curcumin encapsulation through tunable crosslinked hydrogel microbeads.

#### 2.2.6. Electrohydrodynamic Atomization (EHDA) Technique

Microencapsulation methods such as extrusion, freeze-drying, and others are commonly used in biomedical applications. However, their use is limited due to the requirement of high temperatures or organic solvents [35]. The electrohydrodynamic atomization (EHDA) technique has emerged as a promising encapsulation alternative that can enhance product fabrication without high temperatures [25]. Also known as electrospraying, EHDA is one of the most notable current microencapsulation techniques [25,36]. Similar to electrospinning, electrospray is a simple and effective method for generating polymeric or fiber-based bioactive nanoparticles. With EHDA, it is possible to overcome the drawbacks of conventional microencapsulation like poor scale-up, low encapsulation efficiency, and particle polydispersity, as indicated in [25]. Furthermore, sustained drug release in the GIT can be readily achieved with the EHDA technique.

**Figure 3:**
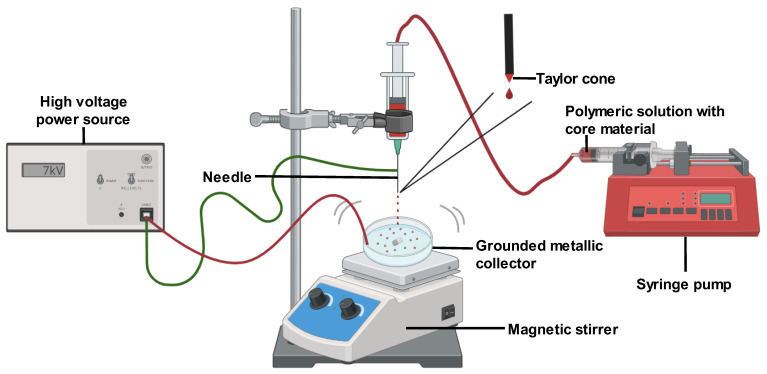
Electrohydrodynamic atomization (EHDA) technique.

Monodispersed particles ranging from microns to hundreds of microns can be produced by EHDA by leveraging electrostatic differences [4,36]. In this method, a syringe is filled with a polymeric solution containing the coating material and core substance and then connected to an infusion pump with a preset regulated flow [36]. A high positive voltage is applied between the needle and a grounded metallic collector, generating an electrostatic field and high voltage on the polymeric solution jet, with negative discharge occurring at the collector [4,25]. Exposure to the electric field deforms the liquid droplet surface at the capillary nozzle tip into a cone shape (Taylor cone) due to internal electrostatic repulsions and external coulombic attraction, from which the jet is ejected. The jets then break into fine droplets due to varicose instability, forming solution jets that are collected as the capsules form [4,36]. Ref. [36] noted that the lower voltage and viscosity encourage highly charged droplets that scatter with solvent evaporation, yielding deposited capsules. A low polymer concentration is needed to destabilize the jet and form tiny droplets. Moreover, low-molecular-weight particles may not provide sufficient viscosity but offer strong inter- and intramolecular forces that must be balanced via surfactant addition [25] (Figure 3).

Several parameters should be considered when preparing microparticles using the EHDA technique. According to [25], the release kinetics of encapsulated core material are influenced by the shape and size of the electrosprayed particles. These are in turn affected by three main factors: solution properties, operational variables, and environmental conditions. Solution properties include viscosity, surface tension, conductivity, polymer concentration, and molecular weight. Operational variables are the distance between the needle tip and collector, flow rate, and applied voltage. Environmental factors comprise humidity, temperature, and airflow. Ref. [25] found that particles tend to have a more spherical versus irregular shape.

Polymer concentration and molecular weight impact the surface tension and viscosity of the polymeric solution, as [25] discussed. High polymer concentrations are needed to form particles from low-molecular-weight polymers, while high-molecular-weight polymers can yield particles even at low concentrations. Solution viscosity is key for optimizing the process. Surface tension also affects droplet formation, with polymers having low surface tension generating smaller droplets and particles [25]. The polymer’s conductivity and solvent are critical for electrospraying since they influence the electrostatic attraction to the collector. Higher conductivity increases Coulombic repulsion forces that compete with solution viscoelastic forces, untangling polymer chains to form smaller particles. Additionally, the applied voltage is crucial for monodisperse particles. Altering the voltage can modify particle morphology; high voltages result in elongated particles, according to [25].

The EHDA technique is a cost-effective, versatile microencapsulation method with significant potential for drug delivery applications, as described by [4]. It is also a simple, environmentally friendly one-step process that does not require organic solvents and high temperatures [4,30,36]. EHDA can encapsulate both lipophilic and lipophobic substances [36]. As indicated in [4,26] and [25], EHDA allows for the formation of small beads with uniform, narrow size distribution, and greater swelling and diffusion rates. This enhances process performance regarding encapsulation efficacy and formulation flexibility compared to spray drying [25,26,36]. EHDA also achieves higher loading efficiency and particle deposition rates [4]. The technique boosts core material bioavailability and solubility while enhancing protection via improved physical and functional properties [4,25]. Microbeads with larger surface-area-to-volume ratios and more inter/intraparticle pores are also ensured with EHDA [25]. Additional benefits include tailored release profiles and masking undesirable chemicals [4,25], indicating the potential for scale-up. Ref. [30] developed EHDA-enabled small peppermint oil-loaded alginate microbeads with good encapsulation efficiency using a simple apparatus. Ref. [4] reported enhanced release profiles for EHDA-produced PLGA microparticles and 95% encapsulation efficacy for curcumin-loaded PLA microcapsules, which also exhibited potent antibacterial and antioxidant activities. PLA microcapsules developed with EHDA displayed high biocompatibility and minimal cytotoxicity [4]. Encapsulation efficiency around 90% was seen for curcumin-loaded zein–chitosan particles using EHDA [4]. Thus, EHDA is an effective, promising technique for encapsulating bioactive compounds like curcumin.

### 2.3. Current Approaches on Encapsulating Curcumin for Drug Delivery

To overcome curcumin limitations resulting in its poor bioavailability and rapid systemic clearance after oral administration, and to fully utilize the therapeutic potential of curcumin, innovative drug delivery systems are required. The encapsulation of curcumin in a protective carrier can enhance its stability, solubility, and absorption while controlling its release rate [37]. Particulate carriers like nanoparticles and microparticles have shown promise for curcumin delivery by increasing surface area, protecting against degradation, bypassing first-pass metabolism, and providing sustained release [3]. While nanoencapsulation has been widely investigated, there are limited studies on formulating curcumin into microbeads using emulsification-based techniques like electrohydrodynamic atomization (EHDA). Compared to nanoparticles, microbeads offer advantages like improved encapsulation efficiency, reduced burst release, and better control over drug release kinetics.

Alginate, a naturally occurring anionic polysaccharide, is an attractive polymer for fabricating drug-loaded microbeads owing to its biocompatibility, low cost, and entrapment efficiency [8]. By optimizing process parameters like voltage, flow rate, and polymer concentration, the size, morphology, drug loading, and release characteristics of alginate microbeads can be tailored using the EHDA technique. Site-specific delivery to the colon can also be achieved by exploiting the colonic microflora-triggered degradation of alginate. Therefore, the proposed research aims to develop curcumin-loaded alginate microbeads using EHDA as an oral delivery system, with enhanced stability, bioavailability, and colon-targeted release intended for therapeutic applications like colon cancer treatment. The systematic optimization and characterization of the microbeads will provide fundamental insights into designing efficacious curcumin delivery systems.

Various drug delivery systems have been explored to overcome the poor water solubility, chemical instability, rapid metabolism, and low bioavailability of curcumin. Encapsulation protects curcumin from degradation, improves its solubility and absorption, and provides controlled release at target sites [38,39]. For instance, the authors of [38] encapsulated curcumin in iron oxide nanoparticles using coprecipitation. The nanocarrier improved curcumin’s solubility and achieved high encapsulation efficiency. The pH-sensitive sustained release of curcumin was demonstrated, with more release at lower pH. This nanoformulation shows promise for targeted curcumin delivery. In another study [39], the authors prepared curcumin-loaded complex coacervates using gum arabic and whey protein nanofibrils. An exceptionally high encapsulation efficiency of 99% was attained, along with enhanced antioxidant activity, versus free curcumin. The coacervates displayed sustained release with more curcumin release under simulated gastric conditions due to weakened electrostatic interactions.

Beyond nanoparticles, polymeric microcapsules also offer advantages like high encapsulation efficiency, tunable drug release profiles, and improved stability. Mai et al. (2017) [40] encapsulated curcumin in poly(lactic acid) (PLA) microcapsules fabricated via electrospraying. Encouragingly, encapsulation efficiency exceeded 95%, and sustained release was maintained for 200 h after a minimal initial burst. Cytotoxicity assays confirmed that microcapsules were nontoxic and highly biocompatible. Silk fibroin nanoparticles developed by [41] delivered encapsulated curcumin in a time-dependent manner to colon cancer cells with enhanced permeability. The nanoparticles provided a slow, sustained release to amplify anticancer effects while reducing side effects. Chitosan–zein nanoparticle carriers synthesized by [42] exhibited a high encapsulation efficiency of 92% and potent cytotoxic activity against neuroblastoma cells. Collectively, these studies demonstrate that nano- and microencapsulation are promising strategies to improve the efficacy and pharmacological properties of curcumin for oral, intravenous, and site-specific delivery. The systematic optimization of formulation parameters and thorough in vitro/in vivo characterization will be imperative to translate these innovative curcumin delivery systems into viable clinical therapeutics.

In addition to the nano- and microencapsulation approaches already discussed, researchers have also explored other techniques like ionotropic gelation to improve the efficacy of curcumin as a therapeutic agent. For example, the authors of [43] encapsulated curcumin in nanoparticles using ionotropic gelation with sodium alginate, achieving high encapsulation efficiency of up to 95%. The nanoparticles displayed minimal dissolution in simulated gastric and intestinal fluid but readily dissolved in simulated colonic fluid. The oral bioavailability of nanoencapsulated curcumin was enhanced five-fold compared to free curcumin. Thus, this formulation shows promise for the targeted delivery and improved efficacy of curcumin for treating colon diseases.

The authors of [44] developed another nanoformulation by encapsulating curcumin in alginate oligosaccharide nanoparticles. The controlled release of curcumin was demonstrated, with 28.9% and 67.5% cumulative release under neutral and acidic conditions, respectively. An excellent trapping efficiency of 91% was also reported. Compared to free curcumin, the nanoparticles showed enhanced absorption by colon cancer cells and improved tumor cell targeting. This highlights the potential of nanoencapsulation to improve the selectivity and anticancer efficacy of curcumin.

Recent studies have evaluated a wide range of encapsulation techniques, carrier materials, and formulations to enhance the therapeutic potential of curcumin. Table 4 provides a comprehensive overview of the various polymers, methods, and formulations developed to effectively deliver curcumin for treating different diseases. Biocompatible polymers like alginate, chitosan, gelatin, and poly(lactic-co-glycolic acid) (PLGA), as well as many formulations such as solid lipid nanoparticles, polymeric micelles, and liposomes, have also been investigated. Encapsulation consistently improves the solubility, stability, sustained release, bioavailability, and pharmacological activity of curcumin, both in vitro and in vivo. As research continues to advance for optimizing and refining curcumin delivery systems, translation from the lab to clinical applications will require a thorough characterization of each formulation’s safety, stability, scalability, and efficacy. Overall, nano- and microencapsulation demonstrate immense potential to finally unlock the full therapeutic potential of curcumin.

## 3. Summary and Conclusions

Curcumin is a natural, affordable, nontoxic polyphenol that is widely available [12]. It is derived primarily from the rhizomes of Curcuma longa, also known as turmeric, which is in the Zingiberaceae family. Curcumin can also be obtained from the root tubers of Curcuma aromatica Salisb., known as wild turmeric [11]. Historically, curcumin was used in ancient China as a medicine to heal pain, inflammation, and other illnesses by promoting blood circulation and removing blood stasis [11]. More recently, curcumin’s potential to prevent and treat cancer has received significant attention in cancer research. Its biological activities are believed to arise from its anti-inflammatory and antioxidant properties, which are important in treating diseases such as leukemia and lymphoma, as well as breast, lung, and gastrointestinal cancers [4,5,11]. Studies show that it can substantially promote apoptosis and inhibit the invasion, metastasis, and angiogenesis of cancer cells, demonstrating its potential for cancer management [4]. Curcumin has proven to be well tolerated at high oral doses with minimal side effects, indicating that it is safe for human use even at high concentrations. However, curcumin suffers from poor absorption, rapid metabolism, and fast elimination, leading to its extremely low bioavailability, aqueous solubility, and limited distribution to target sites, consequently hampering its clinical application for treating various diseases [7,8,37]. This review aims to examine curcumin and formulations that can improve its bioavailability and stability by incorporating it into an appropriate oral drug delivery system targeting the gastrointestinal tract, specifically the colon. Controlled drug delivery systems primarily seek to reduce dosing frequency and enhance efficacy by localizing drugs to their site of action while achieving uniform distribution and improved bioavailability. Moreover, by delivering high local concentrations, low systemic levels can be maintained, minimizing side effects and improving patient compliance [19]. Various encapsulation methods for curcumin fall into three main categories: physical, chemical, and physicochemical methods. Examples include in situ polymerization (chemical), spray chilling and fluidized bed coating (physical), and coacervation (physicochemical) [20,25].

Many curcumin formulations have already undergone clinical trials over the years, with few of them falling within the scope of this review and currently marketed in products like Theracurmin^®^ HP and Longvida^®^ solid lipid nanoparticles [79]. The excellent enhanced, sustained, and targeted delivery that can only be achieved by using polymers gives polymeric micro- and nanoformulations a clear advantage over other techniques. Not only do these clinical trials show the noticeable positive effects of these formulations in treating various diseases, but their safety profiles also appear to be excellent; thus, the continuous development of such supplements is an increasing trend, and more sophisticated and costly manufacturing processes are no longer off the table to harvest more positive clinical outcomes in the long run.

## Figures and Tables

**Table 1:** Comparing different microencapsulation techniques for curcumin.

Technique	Scale	Cost	Mildness	Release Control	Stability
Spray drying	Industrial	Low	Low (high temps)	Moderate	Moderate
Spray chilling	Industrial	Low	High	Moderate	Moderate
Fluidized bed coating	Industrial	Moderate	High	Moderate	Moderate
Coacervation	Small to moderate	Low	High	High	High
Ionotropic gelation	Small to moderate	Low	High	High	Moderate

**Table 2:** Polymers and materials for curcumin microencapsulation.

Polymer/Material	Biocompatibility	Degradation	Mechanical Properties	Encapsulation Suitability
Alginate	High	Slow	Brittle	Coacervation, ionotropic gelation
Chitosan	High	Slow	Brittle	Ionotropic gelation
Cellulose	High	Slow	Brittle	Spray drying, spray chilling
Starch	High	Moderate	Brittle	Spray drying, spray chilling, fluidized bed coating
Whey protein	High	Fast	Flexible	Spray drying, spray chilling
Lecithin	High	Fast	Flexible	Spray drying, spray chilling, coacervation
Gelatin	Moderate	Fast	Flexible	Coacervation, ionotropic gelation
Poly(lactic-co-glycolic) acid	Moderate	Moderate	Rigid	Spray drying, spray chilling, fluidized bed coating
Polycaprolactone	High	Slow	Flexible	Spray drying, spray chilling

**Table 3:** Factors influencing curcumin encapsulation efficiency and release kinetics with different microencapsulation techniques.

Factor	Impact
Polymer type	Affects encapsulation efficiency and release rate
Polymer molecular weight	Higher MW results in a slower release
Polymer concentration	Higher concentration improves encapsulation and extends release
Curcumin loading	Higher loading decreases encapsulation efficiency
Emulsifier type and concentration	Impacts encapsulation efficiency and release rate
Crosslinking density (ionotropic gelation)	Higher density prolongs the release
Inlet temperature (spray drying)	Higher temperature decreases encapsulation efficiency
Flow rate	A slower rate improves encapsulation efficiency
Stirring/homogenization rate	A faster rate gives smaller and more uniform particles
pH	Impacts polymer–curcumin interactions and release

**Table 4:** Summary of curcumin-loaded formulation technique using various polymers as a drug delivery system.

Types of Particles	Formulation Technique	Polymer(s)	Concentration of Polymer(s)	Target System	Findings	Reference
Nanoparticle	Solution-enhanced dispersion by supercritical CO_2_	Silk fibroin powder	N/A	Intestine (Colon)	Permeability, retention effect, and intracellular uptake efficiency have shown to be improved in a time-dependent manner with this formulation, thus enhancing the inhibitory activity of curcumin on CRC (>98%). Moreover, it has been found that at a concentration of ~10 μg/mL, toxicity on healthy human colon mucosal epithelial cells is reduced, mainly owing to its sustained drug release mechanism.	[41]
Nanoparticle	Oil-in-water emulsion-solvent evaporation technique	PEGylated (polyethylene glycol)-PLGA	N/A	Intestine (Colorectal)	Controlled drug release and higher intracellular drug concentrations are observed with this formulation, leading to an improved anticancer action toward the colorectal cancer cells.	[45]
Microcapsule	Electrospray	PLA	1–7%	Intestine	High encapsulation efficiency of more than 95% is achieved with this formulation, as well as the sustained drug release system for up to 200 h. Moreover, a minimal cytotoxic effect and excellent biocompatibility are observed with this formulation too.	[40]
Solid lipid microparticles incorporated in cold-set emulsion-filled gels		Xanthan gum and soy protein isolate	Xanthan gum (0.1% *w*/*v*) Soy protein isolate (15% *w*/*v*)		Curcumin is successfully encapsulated in this formulation with enhanced stability.	[46]
Microparticles	Ionotropic gelation	Chitosan and sodium alginate	Chitosan (0.47 mg/mL) Sodium alginate (0.63 mg/mL)	Topical	This formulation has enhanced curcumin’s wound-healing effect with an encapsulation efficiency of 75%. Moreover, the safety of curcumin has also been confirmed at a concentration of less than 25 µg/mL. Sustained drug release is achieved with this formulation as well, proving the potential use of this biodegradable formulation to deliver curcumin topically.	[47]
Nanoparticle	Freeze-drying method	Polyvinyl alcohol (PVA)	0.1%	Intestine (Colon)	The encapsulation efficiency and aqueous solubility of curcumin are improved.	[48]
Nanoparticles	Emulsification and crosslinking process	Sodium alginate	0.6 mg/mL	Prostate	The slow release of curcumin from the nanoparticle is demonstrated, as well as a higher uptake by the cell. Moreover, its cytotoxic action toward the prostate cancer cells is also observed, without the presence of hemodialysis, proving its safety to be administered intravenously.	[49]
Nanoparticles	Co-precipitation approach	Sodium alginate	40%	GIT	The fabricated nanoparticles exhibited an excellent sustained drug release profile in a pH-sensitive manner, with a prolonged duration of action. Owing to its high entrapping efficiency and pH-sensitive drug release property, it has been deemed as one of the potential approaches for targeted drug delivery systems.	[38]
Nanoparticles	Oil-in-water emulsion–solvent evaporation technique and Freeze-drying	Ovalbumin and κ-carrageenan	0.05%	GIT	An excellent encapsulation efficiency is observed with this formulation whilst the stability against heat and light has improved. Moreover, an increased antioxidation event is observed too. A gradual release of curcumin from the formulation is also demonstrated, hence improving its bioavailability as well as stability in an acidic environment.	[50]
Liposome	N/A	1,2-distearoyl-sn-glycero-3-phosphoethanolamine-N-[amino(PEG_2000_)] (DSPE-PEG_2000_)	2 mM	Intestine (Colon)	A better inhibitory action toward the colon cancer cells is observed with this formulation.	[51]
Hydrogel (Microgels)	Cold gelation or GDL-induced gel formation?	Whey protein aggregates and k-carrageenan	Whey protein aggregates (12.6% *w*/*w*) k-carrageenan (0.1 and 0.55%, *w*/*w*)	Intestine (Colon)	The high trapping efficiency of curcumin is demonstrated with this formulation. With this formulation, curcumin is also protected from degradation and release in the upper GIT, hence, it is deemed as a potential drug delivery system that is targeted to the colon.	[52]
Nanoparticle	Electrospray	Chitosan, zein, and piperine	Chitosan (2%) Zein (5%) Piperine (1.21 mg/mL)	Neuroblastoma cells	High trapping efficiency of curcumin of 92% is observed with this formulation, as well as, a great cytotoxic action toward the neuroblastoma cells at a concentration of 10–25 µg/mL.	[42]
Micelle	Nanoprecipitation	Methoxy PEG-poly(caprolactone)	-	Intestine (Colon)	This formulation has achieved a synergistic effect on killing the colon cancer cells.	[53]
Liposome/Nanofiber mats	Ethanol injection method/ Electrospinning technique	Gelatin and zein	Gelatin (20% *w*/*w*) Zein solutions (20% *w*/*w*)	-	Improved stability and a high encapsulation efficiency of approximately 90% of curcumin are achieved with this formulation. Moreover, a slow release of curcumin from the formulation is also observed.	[54]
Nanoparticles	Co-precipitation OR layer-by-layer coating	Sodium alginate and chitosan	Sodium alginate (20 mg/mL) Chitosan (10 mg/mL)	Breast	Sustained drug release and improved cellular uptake efficiency, as well as superior cytotoxic action toward cancer cells, are achieved with this formulation; hence, this formulation is deemed one of the most promising approaches to delivering drugs for cancer treatment.	[55]
Liposome	W/O emulsion mediated film dispersion method	DSPE-PEG_2000_	-	Intestine (Colon)	It has been shown that this formulation can improve the cellular uptake and lysosome escape of curcumin. This formulation led to better inhibition of cell proliferation than the free curcumin in colon cancer cells.	[56]
Microencapsulated matrix/ Nanofibrous scaffold	Freeze gelation	Chitosan and sodium alginate	-	Topical (Wound)	Great cytotoxic action toward cancer cells is achieved with this formulation. Moreover, the formulation is also found to be stable against enzymatic degradation.	[57]
Micelles	-	Sodium alginate	-	-	Controlled drug release is demonstrated with this formulation for up to 5 h, under physiological conditions. Its safety to be used in humans is also demonstrated by the absence of aggregation and cell hemolysis. Rapid cellular uptake is also observed. Hence, it is deemed a safe and efficient drug delivery system for curcumin.	[58]
Complex coacervates	Complex coacervation method	Gum arabic and whey protein nanofibrils	-	GIT	High encapsulation efficiency of up to 99% is observed with this formulation. Moreover, an excellent antioxidation activity and controlled drug release mechanism are also observed under simulated GIT conditions.	[39]
Complexes	Solvent-free pH shifting method	Whey protein isolate	20 mg/mL	GIT	It significantly improved the aqueous solubility, chemical stability, and antioxidation effect of curcumin. A controlled drug release was also observed.	[59]
Nanoparticle	Ionic gelation technique	Chitosan and sodium alginate	Chitosan (0.05% *w*/*v*) Alginate (0.025% *w*/*v*)	-	A drug loading efficiency of up to approximately 90% was achieved. The sustain-release of curcumin following the zero-order kinetics was also achieved with this formulation.	[60]
Nanoparticles	Ionotropic gelation technique	Sodium alginate	1%	Intestine (Colon)	An improved trapping efficiency, up to 95%, was observed in this formulation. Moreover, it has been demonstrated that the dissolution of prepared nanoparticles is extremely low in simulated gastric and intestinal fluid, where most of the drug is released in simulated colonic fluid. Moreover, the oral bioavailability of curcumin is also found to be enhanced by 5-fold after the encapsulation.	[43]
Sponge (Wound dressing)	Freeze-drying	Ring-shaped β-cyclodextrin, chitosan, and sodium alginate	Chitosan (1%) Alginate (1%)	Topical	The drug release, rate of degradation, and water uptake profiles of the formulation were found to be suitable as a wound-dressing formulation, where this cutaneous formulation facilitated the wound-healing process.	[61]
Nanoparticles/Wound dressing	Emulsification–diffusion method	Polycaprolactone and sodium alginate	Polycaprolactone (2% *w*/*v*) Sodium alginate (4% *w*/*v*)	Topical	It has a good absorbency ability to remove the possible exudates, with high mechanical strength. Gradual release of curcumin is also achieved with delayed degradation.	[62]
Microparticle	Spray drying	Gum arabic	1% *w*/*v*		The microcapsules prepared with gum arabic and sodium alginate have very rough surfaces, whilst microcapsules prepared with chitosan have a smoother surface. The release profile is similar among the three formulations, where the total release times are 4 h, 2 h, and 35 min, in microparticles prepared with gum arabic, sodium alginate, and chitosan, respectively. High trapping efficiency up to 93.8% to 97.6% is also achieved.	[63]
		Sodium alginate				
		Chitosan				
Biopolymer composite film	Mechanical blending and casting method	Bacterial cellulose, alginate, and gelatin	-	Topical	The fabricated film showed the potent anticancer effect against the oral cancer cells, with mucoadhesion time at 0.5 to 6 h to porcine mucosa, under the artificial saliva condition.	[64]
Microfibers	Ionotropic gelation method	Sodium alginate and gelatin	Sodium alginate (10–100%) Gelatin (10–90%)	Topical	Prolonged release of curcumin, up to 85% in 72 h, was achieved. It is deemed a potential drug delivery system for wound management, owing to its significant wound-healing profile.	[65]
Microspheres	-	Chitosan	-	GIT	A pH-sensitive swelling profile and controlled drug release mechanism are seen with the prepared microspheres. The formulation has lesser toxicity effects and its safety has been proved in the study after being incubated against the normal cell line. Hence, it is considered a potential approach for the controlled drug delivery system.	[66]
Microparticles	Polyelectrolyte complexation and ionic crosslinking	i-carrageenan, gellan and chitosan	-	Intestine (Colon)	High encapsulation efficiency of 85.75% to 97.25% was observed with this formulation. The potential use of this oral formulation as the controlled-release colon-targeted drug delivery was also demonstrated in this study, where curcumin within the microparticles was found to be able to overcome the gastric barrier without being degraded.	[67]
Nanosphere	Ionotropic gelification	Sodium alginate, chitosan, β-cyclodextrin, and PEG	-	Breast	High encapsulation efficiency and slow release of curcumin were observed with this formulation. Moreover, its absorption was also improved with this formulation, while the cell proliferation of the breast cancer cells was significantly reduced after the treatment.	[68]
Microparticles	Vacuum spray drying	Jelly fig extract and dicalcium phosphate hydroxide	Jelly fig extract (1.5–4.5% *w*/*w*) Dicalcium phosphate hydroxide (0.075% *w*/*w*)	GIT	High encapsulation efficiency up to 91.56% is achieved with this formulation. Moreover, the formulation also exhibits an improved antioxidation stability profile and a cumulative drug release of approximately 95.34% in 1 d.	[69]
Microbeads	In situ ion-exchange followed by simple ionotropic gelation technique	Sodium alginate	-	Intestine	Extended drug release is seen with this formulation, showing that it is a potential approach to delivering curcumin.	[70]
Nanoparticles/ Scaffolds	-	Collagen, alginate, and PEG_6000_	Collagen (1%) Alginate (2%) PEG_6000_ (50%)	Topical	Owing to its improved permeability, bioavailability, and aqueous solubility achieved with this formulation, it facilitates the wound-healing process. Moreover, a good curcumin encapsulation profile was also observed.	[71]
Hydrogel beads	Ionotropic gelation method	Sodium alginate	2%	GIT	A controlled release of curcumin was achieved with this formulation under simulated GIT conditions.	[72]
Nanoparticle	Nanoprecipitation	poly(D,L-lactide)	5 mg/mL	Intestine (Colon)	This formulation revealed an improved curcumin’s biodistribution and anticancer profiles against the human colon cancer model.	[73]
Nanoparticles	-	Alginate oligosaccharides	1% *w*/*v*	Intestine (Colon)	Encapsulation efficiency was 91%. Controlled drug release was achieved with this formulation but was partially inhibited under neutral conditions. The absorption of the fabricated nanoparticles by colon cancer cells was better than the free curcumin, thus improving its tumor cell target efficiency. Findings suggested that this formulation might be a promising approach in the treatment of colon cancer.	[44]
Hydrogel	Ionic gelation technique	Alginate and chitosan	-	Lung and breast	It has been demonstrated that this formulation has a better anticancer profile.	[74]
Microsphere/Microbead	Emulsion-templated ionic gelation	Sodium alginate/Chitosan	Sodium alginate (3%)	Breast	A pH-sensitive drug delivery system has been achieved with this formulation. Moreover, its anticancer activity has improved too.	[75]
Polymer Encapsulated Liposomes	pH-driven method after extrusion of liposomes	PEG, Eudragit^®^S100	-	Intestines	PEG reduced the bile effect on digestion, and Eudragit^®^S100 blocked the early release of the encapsulated drug in the mouth and stomach.	[76]
Composite film	Physical crosslinking	Poly(vinylalcohol)(PVA)/gelatin	-	Wounded skin	Rapid wound healing was observed, and histopathology showed ordered collagen deposition and angiogenesis with fibroblast formation and increased expression of proinflammatory cytokines	[77]
hydrogel	Thin film hydration for curcumin micelles, solvent casting method for the hydrogel formation	Pluronic F-68 for curcumin micelles, Chitosan and carboxymethyl cellulose for the hydrogel	-	Wounded skin	The hydrogel was biocompatible both using 3T3-L1 fibroblasts cell and in vivo, and improved wound healing observed by histopathology	[78]

## Data Availability

Data will be provided upon request.

## References

[1] Jamwal R. (2018). Bioavailable curcumin formulations: A review of pharmacokinetic studies in healthy volunteers. J. Integr. Med..

[2] Rathore S., Mukim M., Sharma P., Devi S., Nagar J.C., Khalid M. (2020). Curcumin: A review for health benefits. Int. J. Res. Rev..

[3] Hassanzadeh K., Buccarello L., Dragotto J., Mohammadi A., Corbo M., Feligioni M. (2020). Obstacles against the Marketing of Curcumin as a Drug. Int. J. Mol. Sci..

[4] Abd El-Hack M.E., El-Saadony M.T., Swelum A.A., Arif M., Abo Ghanima M.M., Shukry M., Noreldin A., Taha A.E., El-Tarabily K.A. (2021). Curcumin, the active substance of turmeric: Its effects on health and ways to improve its bioavailability. J. Sci. Food Agric..

[5] Hewlings S.J., Kalman D.S. (2017). Curcumin: A Review of Its Effects on Human Health. Foods.

[6] Mansouri K., Rasoulpoor S., Daneshkhah A., Abolfathi S., Salari N., Mohammadi M., Rasoulpoor S., Shabani S. (2020). Clinical effects of curcumin in enhancing cancer therapy: A systematic review. BMC Cancer.

[7] Ojo O.A., Adeyemo T.R., Rotimi D., Batiha G.E.-S., Mostafa-Hedeab G., Iyobhebhe M.E., Elebiyo T.C., Atunwa B., Ojo A.B., Lima C.M.G. (2022). Anticancer properties of curcumin against colorectal cancer: A review. Front. Oncol..

[8] Lopresti A.L. (2018). The problem of curcumin and its bioavailability: Could its gastrointestinal influence contribute to its overall health-enhancing effects?. Adv. Nutr..

[9] Sharifi-Rad J., Rayess Y.E., Rizk A.A., Sadaka C., Zgheib R., Zam W., Sestito S., Rapposelli S., Neffe-Skocińska K., Zielińska D. (2020). Turmeric and its major compound curcumin on health: Bioactive effects and safety profiles for food, pharmaceutical, biotechnological and medicinal applications. Front. Pharmacol..

[10] Fuloria S., Mehta J., Chandel A., Sekar M., Rani N., Begum M.Y., Subramaniyan V., Chidambaram K., Thangavelu L., Nordin R. (2022). A Comprehensive Review on the Therapeutic Potential of Curcuma longa Linn. in Relation to its Major Active Constituent Curcumin. Front. Pharmacol..

[11] Peng Y., Ao M., Dong B., Jiang Y., Yu L., Chen Z., Hu C., Xu R. (2021). Anti-inflammatory effects of curcumin in the inflammatory diseases: Status, limitations and countermeasures. Drug Des. Dev. Ther..

[12] Wang Y., Tang Q., Duan P., Yang L. (2018). Curcumin as a therapeutic agent for blocking NF-κB activation in ulcerative colitis. Immunopharmacol. Immunotoxicol..

[13] Rattis B.A., Ramos S.G., Celes M. (2021). Curcumin as a Potential Treatment for COVID-19. Front. Pharmacol..

[14] Dou H., Shen R., Tao J., Huang L., Shi H., Chen H., Wang Y., Wang T. (2017). Curcumin Suppresses the Colon Cancer Proliferation by Inhibiting Wnt/beta-Catenin Pathways via miR-130a. Front. Pharmacol..

[15] Simental-Mendía L.E., Caraglia M., Majeed M., Sahebkar A. (2017). Impact of curcumin on the regulation of microRNAs in colorectal cancer. Expert Rev. Gastroenterol. Hepatol..

[16] Zhu M., Zheng Z., Huang J., Ma X., Huang C., Wu R., Li X., Liang Z., Deng F., Wu J. (2019). Modulation of miR-34a in curcumin-induced antiproliferation of prostate cancer cells. J. Cell. Biochem..

[17] Yashika U., Manoj K.S., Kriti D. (2019). Formulation and evaluation of microbeads for colon targeted drug delivery using natural polymer. World J. Pharm. Med. Res..

[18] Łętocha A., Miastkowska M., Sikora E. (2022). Preparation and characteristics of alginate microparticles for food, pharmaceutical and cosmetic applications. Polymers.

[19] Mullaicharam B., Alka A.J., Sushama P. (2021). Formulation of micro beads: A review. Int. J. Pharm. Sci. Res..

[20] Garg A., Chhipa K., Kumar L. (2018). Microencapsulation techniques in pharmaceutical formulation. Eur. J. Pharm. Med. Res..

[21] Prabhuraj R., Bomb K., Srivastava R., Bandyopadhyaya R. (2020). Selection of superior targeting ligands using PEGylated PLGA nanoparticles for delivery of curcumin in the treatment of triple-negative breast cancer cells. J. Drug Deliv. Sci. Technol..

[22] Chen Y., Chen C., Zhang X., He C., Zhao P., Li M., Fan T., Yan R., Lu Y., Lee R.J. (2020). Platinum complexes of curcumin delivered by dual-responsive polymeric nanoparticles improve chemotherapeutic efficacy based on the enhanced anti-metastasis activity and reduce side effects. Acta Pharm. Sin. B.

[23] Lin X., Wang Q., Du S., Guan Y., Qiu J., Chen X., Yuan D., Chen T. (2023). Nanoparticles for co-delivery of paclitaxel and curcumin to overcome chemoresistance against breast cancer. J. Drug Deliv. Sci. Technol..

[24] da Silva Feltrin F., Agner T., Sayer C., Lona L.M.F. (2022). Curcumin encapsulation in functional PLGA nanoparticles: A promising strategy for cancer therapies. Adv. Colloid Interface Sci..

[25] Coelho S.C., Estevinho B.N., Rocha F. (2021). Encapsulation in food industry with emerging electrohydrodynamic techniques: Electrospinning and electrospraying—A review. Food Chem..

[26] Azad A.K., Al-Mahmood S.M.A., Chatterjee B., Wan Sulaiman W.M.A., Elsayed T.M., Doolaanea A.A. (2020). Encapsulation of black seed oil in alginate beads as a ph-sensitive carrier for intestine-targeted drug delivery: In vitro, in vivo and ex vivo study. Pharmaceutics.

[27] Choudhury N., Meghwal M., Das K. (2021). Microencapsulation: An overview on concepts, methods, properties and applications in foods. Food Front..

[28] Almoustafa H.A., Alshawsh M.A., Chik Z. (2017). Technical aspects of preparing PEG-PLGA nanoparticles as carrier for chemotherapeutic agents by nanoprecipitation method. Int. J. Pharm..

[29] Kahraman E., Güngör S., Özsoy Y. (2017). Potential enhancement and targeting strategies of polymeric and lipid-based nanocarriers in dermal drug delivery. Ther. Deliv..

[30] Azad A.K., Al-Mahmood S.M.A., Kennedy J.F., Chatterjee B., Bera H. (2021). Electro-hydrodynamic assisted synthesis of lecithin-stabilized peppermint oil-loaded alginate microbeads for intestinal drug delivery. Int. J. Biol. Macromol..

[31] Dhivya S., Rajalakshmi A. (2018). A Review on the preparation methods of Curcumin Nanoparticles. Pharma Tutor.

[32] Sun M., Su X., Ding B., He X., Liu X., Yu A., Lou H., Zhai G. (2012). Advances in nanotechnology-based delivery systems for curcumin. Nanomedicine.

[33] Patil P., Karnavat D., Chavan S. (2018). Review on fluidized bed granulation and coating using fluidized bed coater. IAJPR.

[34] Sacco P., Pedroso-Santana S., Kumar Y., Joly N., Martin P., Bocchetta P. (2021). Ionotropic gelation of chitosan flat structures and potential applications. Molecules.

[35] Niamah A.K., Al-Sahlany S.T.G., Ibrahim S.A., Verma D.K., Thakur M., Singh S., Patel A.R., Aguilar C.N., Utama G.L. (2021). Electro-hydrodynamic processing for encapsulation of probiotics: A review on recent trends, technological development, challenges and future prospect. Food Biosci..

[36] Radünz M., Camargo T.M., dos Santos Hackbart H.C., Blank J.P., Hoffmann J.F., Stefanello F.M., da Rosa Zavareze E. (2021). Encapsulation of broccoli extract by electrospraying: Influence of in vitro simulated digestion on phenolic and glucosinolate contents, and on antioxidant and antihyperglycemic activities. Food Chem..

[37] Ahmad R.S., Hussain M.B., Sultan M.T., Arshad M.S., Waheed M., Shariati M.A., Plygun S., Hashempur M.H. (2020). Biochemistry, Safety, Pharmacological Activities, and Clinical Applications of Turmeric: A Mechanistic Review. Evid. Based Complement. Altern. Med..

[38] Manatunga D.C., de Silva R.M., de Silva K.N., de Silva N., Bhandari S., Yap Y.K., Costha N.P. (2017). pH responsive controlled release of anti-cancer hydrophobic drugs from sodium alginate and hydroxyapatite bi-coated iron oxide nanoparticles. Eur. J. Pharm. Biopharm..

[39] Mohammadian M., Salami M., Alavi F., Momen S., Emam-Djomeh Z., Moosavi-Movahedi A.A. (2019). Fabrication and characterization of curcumin-loaded complex coacervates made of gum Arabic and whey protein nanofibrils. Food Biophys..

[40] Mai Z., Chen J., He T., Hu Y., Dong X., Zhang H., Huang W., Ko F., Zhou W. (2017). Electrospray biodegradable microcapsules loaded with curcumin for drug delivery systems with high bioactivity. RSC Adv..

[41] Xie M., Fan D., Li Y., He X., Chen X., Chen Y., Zhu J., Xu G., Wu X., Lan P. (2017). Supercritical carbon dioxide-developed silk fibroin nanoplatform for smart colon cancer therapy. Int. J. Nanomed..

[42] Baspinar Y., Ustundas M., Bayraktar O., Sezgin C. (2018). Curcumin and piperine loaded zein-chitosan nanoparticles: Development and in-vitro characterisation. Saudi Pharm. J..

[43] Govindaraju R., Karki R., Chandrashekarappa J., Santhanam M., Shankar A.K., Joshi H.K., Divakar G. (2019). Enhanced water dispersibility of curcumin encapsulated in alginate-polysorbate 80 nano particles and bioavailability in healthy human volunteers. Pharm. Nanotechnol..

[44] Liu C., Jiang F., Xing Z., Fan L., Li Y., Wang S., Ling J., Ouyang X.-K. (2022). Efficient delivery of curcumin by alginate oligosaccharide coated aminated mesoporous silica nanoparticles and in vitro anticancer activity against colon cancer cells. Pharmaceutics.

[45] Lotfi-Attari J., Pilehvar-Soltanahmadi Y., Dadashpour M., Alipour S., Farajzadeh R., Javidfar S., Zarghami N. (2017). Co-delivery of curcumin and chrysin by polymeric nanoparticles inhibit synergistically growth and hTERT gene expression in human colorectal cancer cells. Nutr. Cancer.

[46] Brito-Oliveira T.C., Bispo M., Moraes I.C.F., Campanella O.H., Pinho S.C. (2017). Stability of curcumin encapsulated in solid lipid microparticles incorporated in cold-set emulsion filled gels of soy protein isolate and xanthan gum. Food Res. Int..

[47] Shahnia M. (2017). Formulating Curcumin in a Biodegradable Polymeric Composite Material: A Step towards Wound Healing Applications. Master’s Thesis.

[48] Dash T.K., Konkimalla V.S.B. (2017). Selection and optimization of nano-formulation of P-glycoprotein inhibitor for reversal of doxorubicin resistance in COLO205 cells. J. Pharm. Pharmacol..

[49] Saralkar P., Dash A.K. (2017). Alginate nanoparticles containing curcumin and resveratrol: Preparation, characterization, and in vitro evaluation against DU145 prostate cancer cell line. AAPS Pharmscitech.

[50] Xie H., Xiang C., Li Y., Wang L., Zhang Y., Song Z., Ma X., Lu X., Lei Q., Fang W. (2019). Fabrication of ovalbumin/κ-carrageenan complex nanoparticles as a novel carrier for curcumin delivery. Food Hydrocoll..

[51] Sesarman A., Tefas L., Sylvester B., Licarete E., Rauca V., Luput L., Patras L., Banciu M., Porfire A. (2018). Anti-angiogenic and anti-inflammatory effects of long-circulating liposomes co-encapsulating curcumin and doxorubicin on C26 murine colon cancer cells. Pharmacol. Rep..

[52] Alavi F., Emam-Djomeh Z., Yarmand M.S., Salami M., Momen S., Moosavi-Movahedi A.A. (2018). Cold gelation of curcumin loaded whey protein aggregates mixed with k-carrageenan: Impact of gel microstructure on the gastrointestinal fate of curcumin. Food Hydrocoll..

[53] Javadi S., Rostamizadeh K., Hejazi J., Parsa M., Fathi M. (2018). Curcumin mediated down—Regulation of αVβ3 integrin and up—Regulation of pyruvate dehydrogenase kinase 4 (PDK4) in Erlotinib resistant SW480 colon cancer cells. Phytother. Res..

[54] Alehosseini A., Gómez-Mascaraque L.G., Martínez-Sanz M., López-Rubio A. (2019). Electrospun curcumin-loaded protein nanofiber mats as active/bioactive coatings for food packaging applications. Food Hydrocoll..

[55] Song W., Su X., Gregory D.A., Li W., Cai Z., Zhao X. (2018). Magnetic alginate/chitosan nanoparticles for targeted delivery of curcumin into human breast cancer cells. Nanomaterials.

[56] Chen Y., Du Q., Guo Q., Huang J., Liu L., Shen X., Peng J. (2019). A W/O emulsion mediated film dispersion method for curcumin encapsulated pH-sensitive liposomes in the colon tumor treatment. Drug Dev. Ind. Pharm..

[57] Mythili Gnanamangai B., Suganya M., Sabarinathan R., Ponmurugan P. (2019). Fabrication of chitosan-alginate microencapsulated curcumin coated scaffold to develop novel cotton crepe bandage. Indian J. Fibre Text. Res. (IJFTR).

[58] Lachowicz D., Karabasz A., Bzowska M., Szuwarzyński M., Karewicz A., Nowakowska M. (2019). Blood-compatible, stable micelles of sodium alginate–curcumin bioconjugate for anti-cancer applications. Eur. Polym. J..

[59] Taghavi Kevij H., Mohammadian M., Salami M. (2019). Complexation of curcumin with whey protein isolate for enhancing its aqueous solubility through a solvent-free pH-driven approach. J. Food Process. Preserv..

[60] Pornpitchanarong C., Sahatsapan N., Rojanarata T., Opanasopit P., Ngawhirunpat T., Patrojanasophon P. (2020). Curcumin-incorporated Thiolated Chitosan/alginate Nanocarriers: Physicochemical Properties and Release Mechanism. Indian J. Pharm. Sci..

[61] Zhao Y., Dai C., Wang Z., Chen W., Liu J., Zhuo R., Yu A., Huang S. (2019). A novel curcumin-loaded composite dressing facilitates wound healing due to its natural antioxidant effect. Drug Des. Dev. Ther..

[62] Guadarrama-Acevedo M.C., Mendoza-Flores R.A., Del Prado-Audelo M.L., Urbán-Morlán Z., Giraldo-Gomez D.M., Magaña J.J., González-Torres M., Reyes-Hernández O.D., Figueroa-González G., Caballero-Florán I.H. (2019). Development and evaluation of alginate membranes with curcumin-loaded nanoparticles for potential wound-healing applications. Pharmaceutics.

[63] Lucas J., Ralaivao M., Estevinho B.N., Rocha F. (2020). A new approach for the microencapsulation of curcumin by a spray drying method, in order to value food products. Powder Technol..

[64] Chiaoprakobkij N., Suwanmajo T., Sanchavanakit N., Phisalaphong M. (2020). Curcumin-loaded bacterial cellulose/alginate/gelatin as a multifunctional biopolymer composite film. Molecules.

[65] Sharma A., Mittal A., Puri V., Kumar P., Singh I. (2020). Curcumin-loaded, alginate–gelatin composite fibers for wound healing applications. 3 Biotech.

[66] Pooresmaeil M., Namazi H. (2020). Facile preparation of pH-sensitive chitosan microspheres for delivery of curcumin; characterization, drug release kinetics and evaluation of anticancer activity. Int. J. Biol. Macromol..

[67] Iurciuc-Tincu C.-E., Atanase L.I., Ochiuz L., Jérôme C., Sol V., Martin P., Popa M. (2020). Curcumin-loaded polysaccharides-based complex particles obtained by polyelectrolyte complexation and ionic gelation. I-Particles obtaining and characterization. Int. J. Biol. Macromol..

[68] Afzali E., Eslaminejad T., Yazdi Rouholamini S.E., Shahrokhi-Farjah M., Ansari M. (2021). Cytotoxicity Effects of Curcumin Loaded on Chitosan Alginate Nanospheres on the KMBC-10 Spheroids Cell Line. Int. J. Nanomed..

[69] Hartini N., Ponrasu T., Wu J.-J., Sriariyanun M., Cheng Y.-S. (2021). Microencapsulation of curcumin in crosslinked jelly fig pectin using vacuum spray drying technique for effective drug delivery. Polymers.

[70] Reddy O.S., Subha M., Jithendra T., Madhavi C., Rao K.C. (2021). Curcumin encapsulated dual cross linked sodium alginate/montmorillonite polymeric composite beads for controlled drug delivery. J. Pharm. Anal..

[71] Mobaraki M., Bizari D., Soltani M., Khshmohabat H., Raahemifar K., Akbarzade Amirdehi M. (2021). The effects of curcumin nanoparticles incorporated into collagen-alginate scaffold on wound healing of skin tissue in trauma patients. Polymers.

[72] Sharma M., Inbaraj B.S., Dikkala P.K., Sridhar K., Mude A.N., Narsaiah K. (2022). Preparation of curcumin hydrogel beads for the development of functional kulfi: A tailoring delivery system. Foods.

[73] Kulbacka J., Wilk K.A., Bazylińska U., Dubińska-Magiera M., Potoczek S., Saczko J. (2022). Curcumin loaded nanocarriers with varying charges augmented with electroporation designed for colon cancer therapy. Int. J. Mol. Sci..

[74] Abbasalizadeh F., Alizadeh E., Bagher Fazljou S.M., Torbati M., Akbarzadeh A. (2022). Anticancer Effect of Alginate-chitosan Hydrogel Loaded with Curcumin and Chrysin on Lung and Breast Cancer Cell Lines. Curr. Drug Deliv..

[75] Boddu A., Obireddy S.R., Zhang D., Rao K., Lai W.F. (2022). ROS-generating, pH-responsive and highly tunable reduced graphene oxide-embedded microbeads showing intrinsic anticancer properties and multi-drug co-delivery capacity for combination cancer therapy. Drug Deliv..

[76] De Leo V., Maurelli A.M., Giotta L., Daniello V., Di Gioia S., Conese M., Ingrosso C., Ciriaco F., Catucci L. (2023). Polymer Encapsulated Liposomes for Oral Co-Delivery of Curcumin and Hydroxytyrosol. Int. J. Mol. Sci..

[77] Rashid N., Khalid S.H., Ullah Khan I., Chauhdary Z., Mahmood H., Saleem A., Umair M., Asghar S. (2023). Curcumin-Loaded Bioactive Polymer Composite Film of PVA/Gelatin/Tannic Acid Downregulates the Pro-inflammatory Cytokines to Expedite Healing of Full-Thickness Wounds. ACS Omega.

[78] Shah S.A., Sohail M., Karperien M., Johnbosco C., Mahmood A., Kousar M. (2023). Chitosan and carboxymethyl cellulose-based 3D multifunctional bioactive hydrogels loaded with nano-curcumin for synergistic diabetic wound repair. Int. J. Biol. Macromol..

[79] Hegde M., Girisa S., BharathwajChetty B., Vishwa R., Kunnumakkara A.B. (2023). Curcumin Formulations for Better Bioavailability: What We Learned from Clinical Trials Thus Far?. ACS Omega.

